# The Palliative Prognostic (PaP) Score without Clinical Evaluation Predicts Early Mortality among Advanced NSCLC Patients Treated with Immunotherapy

**DOI:** 10.3390/cancers14235845

**Published:** 2022-11-27

**Authors:** Andrea De Giglio, Elisa Tassinari, Arianna Zappi, Alessandro Di Federico, Barbara Lenzi, Francesca Sperandi, Barbara Melotti, Francesco Gelsomino, Marco Maltoni, Andrea Ardizzoni

**Affiliations:** 1 Department of Experimental, Diagnostic and Specialty Medicine, University of Bologna, 40138 Bologna, Italy; 2 Medical Oncology, IRCCS Azienda Ospedaliero-Universitaria di Bologna, 40138 Bologna, Italy

**Keywords:** non-small cell lung cancer, immunotherapy, early mortality, prognostic factors, PaP score, LIPI score

## Abstract

**Simple Summary:**

The acceptable safety profile of immunotherapy may affect the risk-benefit ratio analysis of treatment prescription near the late stage of life for advanced non-small cell lung cancer patients. The aim of our retrospective study was to describe the clinical characteristics of patients receiving immunotherapy in the last stages of life and to evaluate the accuracy in predicting short-time mortality of LIPI and PaPwCPS scores. Our findings demonstrated an increased tendency in immunotherapy use in during the last month of life. In this context, a laboratory and clinical score such as the PaPwCPS may improve the physician’s ability to predict early mortality for immunotherapy-eligible patients.

**Abstract:**

Background: An acceptable risk-benefit ratio may encourage the prescription of immune checkpoint inhibitors (ICI) near the late stage of life. The lung immune prognostic index (LIPI) was validated in advanced non-small cell lung cancer (NSCLC) patients treated with ICIs. The palliative prognostic (PaP) score without clinical prediction of survival (PaPwCPS) predicts early mortality probability in terminal cancer patients. Methods: We performed a retrospective study including 182 deceased advanced NSCLC patients, treated with single-agent ICI at our Institution. Two prognostic categories of high and low mortality risk were identified through ROC curve analysis for PaPwCPS and LIPI scores. Results: Most were >65 years of age (68.3%) and received second-line ICI (61.2%). A total of 29 (15.9%) and 131 (72.0%) patients died within 30 and 90 days from treatment start, respectively. A total of 81 patients (44.5%) received ICI during the last month of life. Baseline PaPwCPS and LIPI scores were assessable for 78 patients. The AUC of ROC curves was significantly increased for PaPwCPS as compared with LIPI score for both 30-day and 90-day mortality. A high PaPwCPS score was associated in multivariate analysis with increased 30-day (HR 2.69, *p* = 0.037) and 90-day (HR 4.01, *p* < 0.001) mortality risk. A high LIPI score was associated with increased 90-day mortality risk (*p* < 0.001). Conclusion: We found a tendency towards ICI prescription near the late stage of life. The PaPwCPS score was a reliable predictor of 30- and 90-day mortality.

## 1. Introduction

The advent of immune checkpoint inhibitors (ICIs) dramatically changed therapeutic algorithms and prognostic outcomes of several advanced tumors. In non-small cell lung cancer (NSCLC), ICIs provided benefits in terms of overall survival and safety profile when compared with standard-of-care chemotherapy as an upfront or subsequent line of treatment [1]. ICIs mainly focus on restoring the host immune system, enabling T lymphocytes to recognize and respond to foreign antigens on cancer cells [2]. Since their introduction in clinical practice, ICIs have been deeply investigated, and clinicians are now more confident in the management of the inflammatory side effects, known as “immune-related adverse events” (irAE). Nevertheless, the large majority of irAE can be classified as mild, thus ICIs are generally considered safe agents [3]. Thanks to their high safety profile, single-agent ICIs are often prescribed in patients not eligible for standard chemotherapy due to comorbidities or with short life expectancies [4]. Notably, programmed death ligand 1 (PD-L1) expression is the only predictive biomarker widely used in clinical practice to predict ICI single-agent efficacy. However, when choosing the best treatment option, oncologists should also consider clinical features and laboratory parameters other than tumor biomolecular characteristics. In this regard, the lung immune prognostic index (LIPI) score has been related to worse outcomes for ICI application and could help in identifying patients unlikely to respond [5,6,7,8]. While LIPI is composed entirely of measurable laboratory parameters, other scores that have mainly been used in different medical settings, also encompass clinical features. Among these, the palliative prognostic (PaP) score has been validated in the setting of palliative care to predict survival in terminal cancer patients. This index includes different factors, such as clinical prediction of survival (CPS), Karnofsky performance status (KPS), anorexia, dyspnea, total white blood count (WBC), and lymphocyte percentage [9]. Thus, patients with advanced tumors were sorted into three prognostic risk categories by comparing 30-day mortality rates [10]. However, CPS could be difficult to assess for novice clinicians, who may overestimate survival expectancy, and is usually not reported in medical records.

The prognostic accuracy of the PaP score without CPS (PaPwCPS) has been validated in a prospective trial among 216 hospitalized patients, particularly in those situations where the clinician’s experience was limited [11]. The PaPwCPS included the five variables of the PaP except for CPS, ranging from 0 to 9 points. The PaPwCPS was significantly more efficacious than the PaP score in predicting 30-day survival (*p* < 0.05) [11].

We therefore studied advanced NSCLC patients treated with single-agent immunotherapy with two aims: to describe clinical characteristics of patients receiving ICIs in the last stages of life and to evaluate the accuracy in predicting short-time mortality of LIPI and PAPwCPS scores in patients still undergoing active cancer therapy.

## 2. Materials and Methods

We performed a monocentric, retrospective, observational study including dead patients affected by advanced non-small cell lung cancer, treated with single-agent immunotherapy between August 2015 and December 2021 at the IRCCS Azienda Ospedaliero-Universitaria of Bologna, Italy. Data were obtained from electronic and paper-based medical records. This study received approval from the local ethics committee (approval no. 2381/2019) and was conducted in accordance with the Declaration of Helsinki (1964).

Patients with unknown data regarding death were excluded from the final analysis. The following variables were registered: age, gender, comorbidities, concomitant medications, tumor histology, biomolecular characteristics, antineoplastic treatments, Eastern Cooperative Oncology Group (ECOG) performance status (PS) at baseline and before the last ICI administration, radiological findings at baseline and during the follow-up, number of metastatic sites, biomolecular characterization, symptoms reported at baseline and before the last ICI, blood tests at baseline and before the last ICI administration, last follow-up, cause of death, date of death.

The main objective of the present study was to describe the clinical characteristics of patients receiving the administration of ICI during the last month of life and of those starting a treatment with ICI during the last month and the last three months of life.

The secondary objective was to retrospectively calculate the LIPI and PaPwCPS scores for each patient at baseline to assess the 30-day and 90-day predictive accuracy for mortality. Each score was not registered in clinical records but calculated a posteriori with reported clinical information and laboratory findings. The LIPI score is a prognostic tool validated in advanced NSCLC patients under ICIs, including the dLNR [(neutrophils/(leukocytes minus neutrophils)] and serum LDH levels [5]. dLNR > 3 and LDH above the upper limit of normal give 1 point each. Based on the score, patients are classified into three prognostic groups: 0 for low-risk, 1 for intermediate risk, 2 for high risk. The PAP score is a prognostic score validated in multiple palliative care settings regardless of histology and consists of 6 variables: clinical prediction score (0–8), dyspnea (0–1), anorexia (0–1.5), KPS (0–2.5), white blood cell count (0–2.5), and lymphocyte rate (0–2.5) [9,10]. The CPS is the prediction of survival performed at baseline by a trained physician in palliative care and cannot be determined within a retrospective study. We considered the PaPwCPS score (0–9 points), which has previously been demonstrated to be accurate, as the PaP score but more discriminating for 30-day survival [11]. Our center adopts the ECOG scale to evaluate the PS of cancer patients. Thus, we converted all the ECOG PS to KPS [12] to calculate the score for each patient.

### Statistical Methods

Clinical and laboratory findings were summarized as continuous and categorical variables and reported as median values and proportions. The *t*-test (ANOVA; Pearson correlation test, if needed) and chi2 test (or Fisher’s exact test, if needed) were performed to compare means and proportions. Overall survival was defined as the intercurrent time between treatment start and death for any cause.

The receiver operating characteristic (ROC) curve analysis defined the area under the curve (AUC) per single score, which discriminates the 30-day and 90-day mortality. The AUCs were successively compared through De Long’s test. The best threshold (top left vs. Youden method) was determined at two time points for the mortality discrimination accuracy.

The PaPwCPS score results were divided into two prognostic categories of high mortality risk (≥3.5 score) and low mortality risk (<3.5 score) according to the best threshold assessed through ROC curves. Analogously, LIPI score was categorized into two prognostic categories of high-intermediate mortality risk (1–2 points) or low mortality risk (0 points) according to the best cut-off assessed through ROC curves.

The Kaplan–Meier method was used to estimate OS and log-rank test to compare OS curves according to prognostic score assessment. The relationship between variables and survival outcome was explored through a univariate and multivariate analysis using a Cox model regression for both OS timepoints (30 days, 90 days). A *p*-value ≤ 0.05 was considered statistically significant. Statistical analyses were accomplished with R-Studio free software, version 1.4.1717, utilizing the following packages: ‘dplyr’, ‘prodlim’, ‘survminer’, ‘survMisc’,’ finalfit’, ‘pROC’, ‘CI’, ‘ggplot2’.

## 3. Results

We retrieved the data of 216 patients consecutively treated with single-agent immunotherapy for advanced NSCLC from August 2015 to December 2021. Overall, 182 deceased patients were included in the final analysis. Among them, the majority were older than 65 years (68.3%) and male (62.3%). A total of 73.8% had nonsquamous histology, and 82.2% presented a good (<2) ECOG PS at baseline. A total of 61.2% were treated with second-line immunotherapy (Table 1).

At baseline, the PaPwCPS score was determinable for 86 patients (47.2%), with a mean of 2.5 points (SD 2.0). LIPI score was determinable in 105 cases (57.7%), of which 41 (39%) had a low score, 41 (39%) had an intermediate score, and 28 (22%) had a high score.

A total of 29 patients (15.9%) died within 30 days from the treatment start. The baseline characteristics were balanced in comparison to patients not deceased within 30 days except for a higher prevalence of ECOG PS ≥ 2 (37.9% vs. 13.9%, *p* = 0.005) and a higher mean PaPwCPS score at baseline (3.6 vs. 2.0, *p* = 0.001) (Table 1).

A total of 131 patients (71.9%) died within 90 days from the treatment start. Compared to patients with OS > 90 days, patients in this subgroup were more likely to have ≥ three metastatic sites (55% vs. 36.5%, *p* = 0.037), an ECOG PS ≥ 2 (21.7% vs. 7.8%, *p* = 0.048), a high mean PaPwCPS score (2.9 vs. 0.8, *p* <0.001), and high (26.9% vs. 7.4%) or intermediate (42.3% vs. 29.6%) LIPI score (*p* = 0.008) (Table 1).

A total of 81 patients (44.5%) received immunotherapy during their last month of life. Patients in this subgroup had a higher mean PaPwCPS score compared to those not receiving immunotherapy during the last month of life (3 vs. 1.6, *p* = 0.001; Table 1).

**Figure 1:**
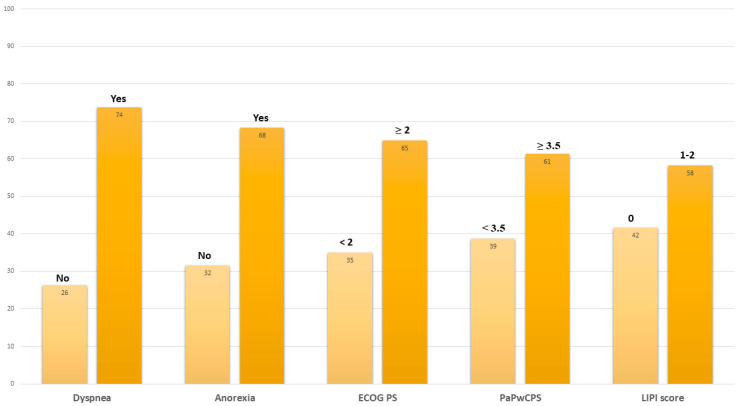
Clinical characteristics registered at last immunotherapy cycle. Abbreviations: ECOG PS, Eastern Cooperative Oncology Group performance status; PaPwCPS, Palli-ative prognostic score without clinical prediction of survival; LIPI, lung immune-prognostic index.

At the moment of the last administration of the ICI, 73.7% of patients complained of dyspnea, 68.4% had anorexia, and 71.1% had an ECOG PS ≥ 2 (Figure 1).

The mean PaPwCPS score was 3.8 (SD 1.8), and 45.8% had a high LIPI score. Comparing the clinical characteristics of patients receiving the last ICI administration to those at the start of immunotherapy, we found a significantly higher rate of dyspnea (*p* = 0.004), anorexia (*p* = 0.008), ECOG PS ≥ 2 (*p* <0.001), and numerically higher mean PAPwCPS (*p* = 0.08) and high-risk LIPI scores (*p* = 0.051), almost reaching the threshold for statistical significance.

### 3.1. Score Predictive Ability Testing

Overall, 78 patients (42.8%) had available clinical and laboratory findings to assess both PaPwCPS and LIPI scores at baseline. All the following analyses were performed on this subgroup of patients.

**Figure 2:**
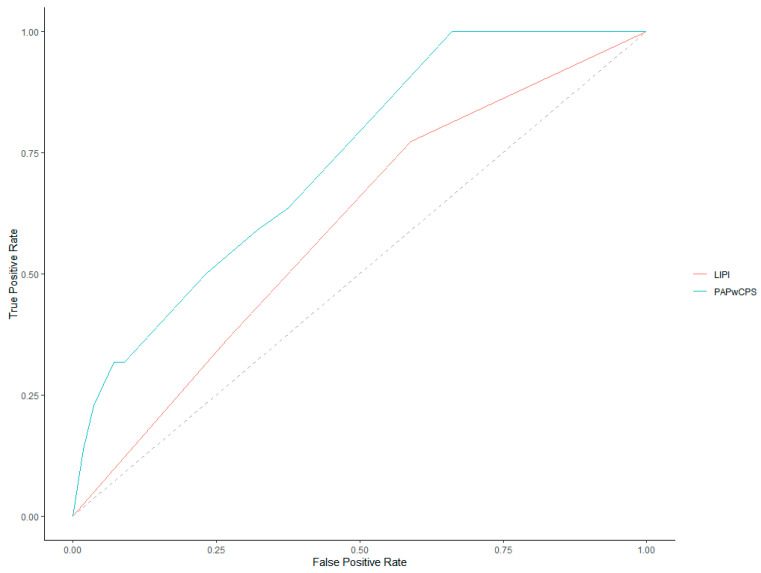
Receiver operating characteristics (ROC) curves for 30-day mortality.

**Figure 3:**
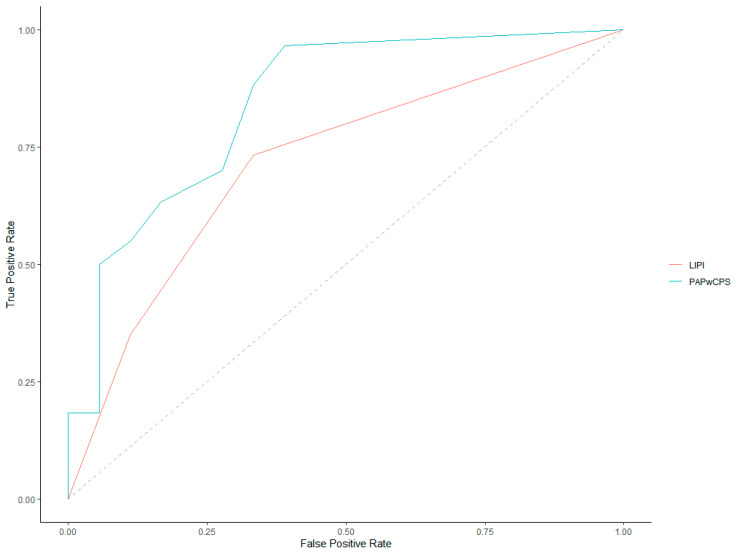
Receiver operating characteristics (ROC) curves for 90-day mortality.

ROC curves for 30-day and 90-day mortality prediction of both LIPI and PaPwCPS scores are represented in Figure 2 and Figure 3.

Investigating the 30-day mortality, the AUC of the ROC curves was 0.59 (95% CI, 0.46–0.72) for LIPI and 0.73 (95% CI, 0.61–0.84) for PaPwCPS. Comparing the AUCs, the PaPwCPS was confirmed to be more accurate (*p* = 0.03). Considering the PaPwCPS score, the best threshold was 0.75 with Youden’s method and 3.25 with the closest top-left point (Table 2).

We set the cut-off at ≥3.5 for an easier clinical applicability. Concerning the LIPI score, the best cut-off value was 0.5 with both Youden’s method and considering the closest top-left point.

Investigating the 90-day mortality, the AUC of the ROC curves was 0.71 (95% CI, 0.59–0.84) for LIPI and 0.84 (95% CI, 0.72–0.95) for PaPwCPS. The latter was found to be significantly more accurate, comparing the AUC of the ROC curves (*p* = 0.03).

### 3.2. Survival Analysis

**Figure 4:**
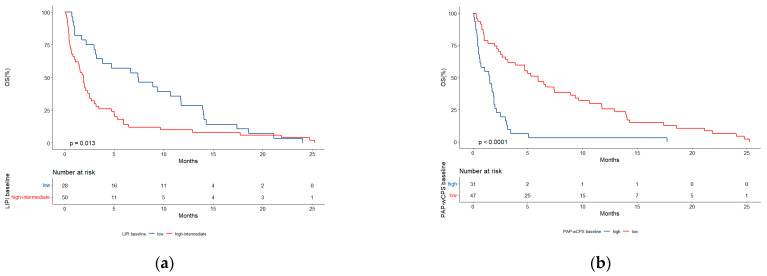
(**a**) Overall survival (OS) according to LIPI score; (**b**) OS according to PaPwCPS score.

Among 182 patients representing the total population analyzed, the median OS was 4.1 months (95% CI, 3.1–5.0). Within the subgroup of score predictive ability testing (N = 78), the median OS was 2.5 months (95% CI, 1.9–4.7). We then analyzed the survival outcomes according to the best cut-offs (LIPI ≥ 1; PaPwCPS ≥ 3.5) that emerged from ROC analyses. Patients with an intermediate-high risk LIPI score had a significantly reduced median OS (1.8 months, 95% CI, 1.0–2.6) in comparison with those with a low-risk LIPI score (7.4 months, 95% CI, 3.1–13.9) (*p* = 0.013) (Figure 3). Similarly, a significantly shorter median OS was observed in patients with high PaPwCPS (1.5 months, 95% CI, 0.5–2.0) as compared to patients with low PaPwCPS (5.9 months, 95% CI, 3.1–9.6) (*p* < 0.0001) (Figure 4).

The Cox regression model showed that a high PaPwCPS score was associated with an increased risk of 30-day mortality in both univariate (HR 2.78, 95% CI, 1.19–6.52, *p* = 0.019) and multivariate (HR 2.69, 95% CI, 1.06–6.83, *p* = 0.037) analyses, including other pivotal variables such as age, gender, line of treatment, and number of metastatic sites (Table 3). Within the same model, high PaPwCPS score was associated with an increased risk of 90-day mortality in the univariate (HR 4.02, 95% CI, 2.32–6.97, *p* < 0.001) and multivariate (HR 4.01, 95% CI, 2.20–7.31, *p* < 0.001) assessment.

A high-intermediate LIPI score was not significantly associated with an increased risk of 30-day mortality in the univariate (HR 2.37, 95% CI, 0.87–6.42, *p* = 0.091) and multivariate (HR 2.76, 95% CI, 0.98–7.82, *p* = 0.055) models, including the same variables mentioned above (Table 4). Conversely, a high-intermediate LIPI score significantly increases the risk of death at 90 days in univariable (HR 2.68, 95% CI, 1.50–4.79, *p* = 0.001) and multivariable (HR 3.22, 95% CI, 1.71–6.07, *p* < 0.001) regression models (Table 4).

## 4. Discussion

The present study confirmed the attitude toward treating patients in the last stages of life in clinical practice, with 15.9% and 71.9% of patients deceased within 30 and 90 days, respectively, from the treatment start, while 44.5% received immunotherapy during the last month of life. In this context, we showed that introducing a laboratory and clinical score such as the PAPwCPS may improve the physician’s ability to predict early mortality for patients eligible for ICI or who are receiving it.

Prescribing systemic treatments near the late stage of life, sometimes called ‘desperation oncology’ [4], may derive from overestimating the possible benefits in a biased risk-benefit balance or from an inadequate evaluation of deteriorated clinical conditions. Beyond the ethical debate about the risk of over- or undertreating cancer patients, especially those naïve to treatments, there is also a considerable risk of financial toxicity affecting both patients and national healthcare systems [13]. The clinical perception of an acceptable tolerability profile may increase the prescription rate of ICI among frail patients. Santini et al. conducted a multicenter retrospective analysis among advanced cancer patients treated with ICI as single agents aimed to investigate their administration during the late stages of life [14]. Among 556 deceased patients, 29.3% of them received ICIs within the last month of life and it was characterized by a significantly higher rate of ECOG PS ≥ 2 (*p* > 0.0001) or high burden of disease (*p* = 0.0266). Furthermore, patients with advanced NSCLC were more likely to start ICI within the last month of life compared to patients affected by other malignancies. Notably, the authors identified an increased trend in ICI prescriptions during the late stages of life within the 2018–2020 period compared to 2014–2017 [14]. Similarly, another observational study found evidence that 67% of 441 advanced cancer patients received ICI during the last 90 days of life and 27% within the last month. They confirmed that a worse ECOG PS (≥3) was associated with 30- or 90-day mortality [15]. Interestingly, ICI administration within the last 90 days of life was related to an increased hospitalization rate, decreased hospice admission, and intrahospital death, leading to additional financial toxicity [15]. Analogously, our experience evidenced a tendency toward treating patients within their last month of life, raising the need for affordable tools to predict early mortality at baseline and during treatment.

Our study confirmed that baseline ECOG PS ≥ 2 was associated with early mortality. Treating patients with ECOG PS ≥ 2 remains an open debate as prospective randomized trials usually exclude these patients, and thus, data mostly derive from retrospective analyses [1]. Still, some phase 2 prospective trials suggested that the toxicity profile of immunotherapy for frail patients is acceptable even if the efficacy may be reduced [16,17]. Interestingly, an observational experience confirmed that a baseline ECOG PS ≥ 2 was associated with shorter survival and was more common in patients receiving ICI during the last month of life [18]. Nevertheless, considering the population of the present study, more than two-thirds of patients who died within 90 days from the treatment start had a baseline ECOG PS of 0–1. In addition, a multicenter study confirmed that ECOG PS should be interpreted cautiously, as only cancer-related PS deterioration should be considered as a predictor of poor clinical prognosis under first-line pembrolizumab for advanced NSCLC [19]. Interestingly, the LIPI score failed to demonstrate a prognostic value among ECOG PS ≥ 2 patients, in terms of OS (*p* = 0.91), within a small subgroup analysis (51/466 patients) [5]. Analogously, LDH, NLR, and dNLR were demonstrated to be predictive biomarkers of survival with moderate accuracy at one year (AUC 0.60, 0.67, and 0.67, respectively) by another retrospective study [20]. These data may indicate that clinical conditions expressed as PS or laboratory test-only scores may not be enough to predict the short-term survival probability.

In this regard, the PAPwCPS score was more accurate in 30-day and 90-day mortality prediction than the laboratory-test-only LIPI score. Maltoni et al. developed the PAP score to estimate the mortality probability among 451 patients in a palliative setting across 14 hospice services [10]. The score predicted 30-day survival probability with a stratification among three risk groups (86.6% vs. 51.6% vs. 16.9% probability). As described, the PAP score originally included a physician-based expectation of survival that is a parameter mainly depending on subjective experience in the field. In one study, eliminating the CPS improved the 30-day accuracy of the score in a prospective multicenter trial [11]. In particular, AUC for 30-day survival was 0.78 (95% CI, 0.7–0.87) for PaPwCPS and 0.73 (95% CI 0.64–0.82) for PaP-score, with a statistically significant difference (*p* < 0.05) [11]. These data have not been confirmed by further studies, which instead show optimal accuracy of the whole score when clinical experience is warranted [21,22]. In addition, outside of a palliative setting, the inclusion of a subjective prediction of mortality could be less reliable for young or treatment-naïve patients. To our knowledge, this is the first study testing the PaPwCPS score in an active anti-cancer treatment setting, which assumes more importance due to the constantly increasing tendency of treating more patients due to the perceived favorable toxicity profile of novel therapies such as ICI. The PaPwCPS score included five items dynamically registered in clinical practice, such as complete blood counts and pivotal clinical information, such as dyspnea, anorexia, and performance status, not depending on biomarkers or other disease-oriented characteristics at baseline. Passaro et al. investigated the inference between baseline clinical factors and 90-day mortality among 321 patients affected by advanced NSCLC with high PD-L1 expression and treated with first-line pembrolizumab [23]. The resulting score showed an AUC of 0.76 (95% CI: 0.70–0.81) for 90-day mortality, but no 30-day mortality analysis was performed. Another observational study explored the factors associated with 60-day mortality among 166 metastatic NSCLC treated with ICI single-agents [24]. Interestingly, laboratory findings (albumin, neutrophils, lymphocytes, C-reactive protein) failed to be independently associated with early death. Conversely, the multivariate model showed that liver involvement, ECOG PS ≥ 2, and a non-smoking history were related to increased 60-day mortality. In our analysis, a high PaPwCPS score was associated with an increased risk of 30-day and 90-day mortality regardless of line of treatment, sex, age, and the number of metastatic sites.

A question of paramount importance is also when to stop the treatment, that is, how to limit therapeutic aggressiveness. Our analysis evidenced that, among patients who received ICI in their last 30 days of life, there was a significant increase in the prevalence of dyspnea, anorexia, and ECOG PS ≥ 2 with a numerical increase in mean PAPwCPS score and high LIPI score rate registered at last ICI administration.

Finally, ICI constitute the first-line backbone for non-oncogene addicted advanced NSCLC. The addition of chemotherapy improved survival outcomes compared with standard chemotherapy, and to date, no head-to-head comparisons have been performed with ICI as single agents, which is of remarkable interest, especially for NSCLCs with high PD-L1 expression (≥50% tumor proportion score) [1,25]. In this setting, the combination strategy may prevent early progression and improve the response rate, and the decision may be driven by clinical conditions or high tumor burden [26]. It is unclear whether the PAPwCPS score may have a role in the decision-making process for therapy selection, but it should be further explored.

The limited sample size and the retrospective design are the main limitations of our study. The variability of clinical reporting and laboratory tests considerably reduced the score testing sample size.

As described, we retrospectively tested the PaPwCPS score using a cut-off not validated by pre-existing literature, but by studying the ROC curve derived from our findings. This threshold should be prospectively verified within a larger population.

## 5. Conclusions

In conclusion, in the context of a confirmed growing attitude in prescribing ICI in cancer patients with poor life expectancy, our study suggests that the use of a score considering clinical and laboratory parameters such as the PaPwCPS is reliable for predicting short-term mortality in advanced NSCLC patients treated with ICI and can aid oncologists in difficult end-of-life treatment decisions, sparing costly and likely useless anti-cancer treatments.

## Figures and Tables

**Table 1:** Baseline characteristics.

			ICI During Last Month of Life			OS ≤ 30 Days			OS ≤ 90 Days		
		Total (%)	No (%)	Yes (%)	*p* Value	No (%)	Yes (%)	*p* Value	No (%)	Yes (%)	*p* Value
Age	≤65	58 (31.7)	32 (31.4)	26 (32.1)	1.000	49 (31.8)	9 (31.0)	1.000	21 (40.4)	37 (28.2)	0.157
	>65	125 (68.3)	70 (68.6)	55 (67.9)		105 (68.2)	20 (69.0)		31 (59.6)	94 (71.8)	
Sex	Female	69 (37.7)	38 (37.3)	31 (38.3)	1.000	56 (36.4)	13 (44.8)	0.513	21 (40.4)	48 (36.6)	0.763
	Male	114 (62.3)	64 (62.7)	50 (61.7)		98 (63.6)	16 (55.2)		31 (59.6)	83 (63.4)	
Smoking status	Current smoker	34 (19.2)	16 (16.7)	18 (22.2)	0.628	28 (18.9)	6 (20.7)	0.870	5 (10.4)	29 (22.5)	0.131
	Former smoker	117 (66.1)	66 (68.8)	51 (63.0)		99 (66.9)	18 (62.1)		37 (77.1)	80 (62.0)	
	Never smoker	26 (14.7)	14 (14.6)	12 (14.8)		21 (14.2)	5 (17.2)		6 (12.5)	20 (15.5)	
Histology	Nonsquamous	135 (73.8)	70 (68.6)	65 (80.2)	0.108	114 (74.0)	21 (72.4)	1.000	39 (75.0)	96 (73.3)	0.959
	squamous	48 (26.2)	32 (31.4)	16 (19.8)		40 (26.0)	8 (27.6)		13 (25.0)	35 (26.7)	
ECOG PS	<2	148 (82.2)	87 (87.0)	61 (76.2)	0.093	130 (86.1)	18 (62.1)	0.005	47 (92.2)	101 (78.3)	0.048
	≥2	32 (17.8)	13 (13.0)	19 (23.8)		21 (13.9)	11 (37.9)		4 (7.8)	28 (21.7)	
Steroid intake	No	137 (74.9)	78 (76.5)	59 (72.8)	0.696	118 (76.6)	19 (65.5)	0.302	42 (80.8)	95 (72.5)	0.331
	Yes	46 (25.1)	24 (23.5)	22 (27.2)		36 (23.4)	10 (34.5)		10 (19.2)	36 (27.5)	
Antibiotic intake	No	163 (89.1)	90 (88.2)	73 (90.1)	0.866	137 (89.0)	26 (89.7)	1.000	48 (92.3)	115 (87.8)	0.534
	Yes	20 (10.9)	12 (11.8)	8 (9.9)		17 (11.0)	3 (10.3)		4 (7.7)	16 (12.2)	
Metastatic sites	≥3	91 (49.7)	44 (43.1)	47 (58.0)	0.064	72 (46.8)	19 (65.5)	0.099	19 (36.5)	72 (55.0)	0.037
	1–2	92 (50.3)	58 (56.9)	34 (42.0)		82 (53.2)	10 (34.5)		33 (63.5)	59 (45.0)	
PD-L1 status	≥50%	52 (47.7)	31 (50.0)	21 (44.7)	0.630	43 (47.8)	9 (47.4)	0.635	16 (57.1)	36 (44.4)	0.493
	0	36 (33.0)	21 (33.9)	15 (31.9)		31 (34.4)	5 (26.3)		8 (28.6)	28 (34.6)	
	1–49%	21 (19.3)	10 (16.1)	11 (23.4)		16 (17.8)	5 (26.3)		4 (14.3)	17 (21.0)	
Line of treatment	≥3	31 (16.9)	17 (16.7)	14 (17.3)	0.676	27 (17.5)	4 (13.8)	0.200	8 (15.4)	23 (17.6)	0.763
	1	40 (21.9)	20 (19.6)	20 (24.7)		30 (19.5)	10 (34.5)		10 (19.2)	30 (22.9)	
	2	112 (61.2)	65 (63.7)	47 (58.0)		97 (63.0)	15 (51.7)		34 (65.4)	78 (59.5)	
PaPwCPS	Mean (SD)	2.5 (2.0)	1.6 (1.8)	3.0 (1.9)	0.001	2.0 (1.9)	3.6 (1.9)	0.001	0.8 (1.3)	2.9 (1.9)	<0.001
dNLR	Mean (SD)	3.2 (2.4)	2.9 (2.3)	3.4 (2.6)	0.166	3.0 (2.3)	3.8 (3.2)	0.159	2.3 (1.7)	3.5 (2.6)	0.010
LIPI	High	23 (21.9)	7 (14.0)	16 (29.1)	0.095	15 (18.1)	8 (36.4)	0.101	2 (7.4)	21 (26.9)	0.008
	Intermediate	41 (39.0)	19 (38.0)	22 (40.0)		32 (38.6)	9 (40.9)		8 (29.6)	33 (42.3)	
	Low	41 (39.0)	24 (48.0)	17 (30.9)		36 (43.4)	5 (22.7)		17 (63.0)	24 (30.8)	

Abbreviations: ICI, immune-checkpoint inhibitor; OS, overall survival; ECOG PS, Eastern Cooperative Oncology Group performance status; PaPwCPS, palliative prognostic score without clinical prediction of survival; dNLR, derived neutrophil-to-lymphocyte ratio; LIPI, lung immune-prognostic index.

**Table 2:** Receiver operating characteristics (ROC) curves analysis.

	30 Days Survival					90 Days Survival				
	AUC	95% CI	Best Threshold (Closest Top-Left)	Best Threshold (Youden Method)	*p* Value (AUC PAP-wCPS vs. AUC LIPI)	AUC	95% CI	Best Threshold (Closest Top-Left)	Best Threshold (Youden Method)	*p* Value (AUC PAP-wCPS vs. AUC LIPI)
PAP-wCPS	0.73	0.61–0.84	3.25	0.75	0.03	0.84	0.72–0.95	0.25	0.75	0.03
LIPI	0.59	0.46–0.72	0.5	0.5		0.71	0.59–0.84	0.5	0.5	

**Table 3:** Univariate and multivariate analyses for 30-day and 90-day survival, including PaPwCPS score. Abbreviations: HR, hazard ratio; n., number.

			30-Days		90-Days	
		All	HR (Univariable)	HR (Multivariable)	HR (Univariable)	HR (Multivariable)
Age	≤65	29 (37.2)	-	-	-	-
	>65	49 (62.8)	0.82 (0.35–1.91, *p* = 0.643)	0.81 (0.34–1.92, *p* = 0.632)	0.85 (0.50–1.45, *p* = 0.554)	1.01 (0.58–1.76, *p* = 0.974)
Sex	Female	26 (33.3)	-	-	-	-
	Male	52 (66.7)	0.52 (0.22–1.21, *p* = 0.128)	0.48 (0.20–1.16, *p* = 0.105)	0.81 (0.48–1.38, *p* = 0.443)	0.68 (0.39–1.20, *p* = 0.188)
Line of treatment	1	26 (33.3)	-	-	-	-
	≥3	10 (12.8)	-	-	1.39 (0.63–3.05, *p* = 0.410)	1.47 (0.64–3.40, *p* = 0.364)
	2	42 (53.8)	0.66 (0.29–1.53, *p* = 0.333)	0.84 (0.34–2.05, *p* = 0.697)	0.95 (0.45–2.01, *p* = 0.885)	1.13 (0.53–2.43, *p* = 0.755)
N. metastatic sites	≥3	42 (53.8)	-	-	-	-
	1–2	36 (46.2)	0.62 (0.26–1.48, *p* = 0.282)	0.68 (0.27–1.71, *p* = 0.407)	0.55 (0.33–0.94, *p* = 0.029)	0.58 (0.33–1.03, *p* = 0.064)
PaPwCPS score	Low	47 (60.3)	-	-	-	-
	High	31 (39.7)	2.78 (1.19–6.52, *p* = 0.019)	2.69 (1.06–6.83, *p* = 0.037)	4.02 (2.32–6.97, *p* < 0.001)	4.01 (2.20–7.31, *p* < 0.001)

**Table 4:** Univariate and multivariate analysis for 30-day and 90-day survival including LIPI score. Abbreviations: HR, hazard ratio; n., number.

		30 Days			90 Days	
		All	HR (Univariable)	HR (Multivariable)	HR (Univariable)	HR (Multivariable)
Age	≤65	29 (37.2)	-	-	-	-
	>65	49 (62.8)	0.82 (0.35–1.91, *p* = 0.643)	0.77 (0.33–1.83, *p* = 0.561)	0.85 (0.50–1.45, *p* = 0.554)	0.86 (0.50–1.48, *p* = 0.583)
Sex	Female	26 (33.3)	-	-	-	-
	Male	52 (66.7)	0.52 (0.22–1.21, *p* = 0.128)	0.46 (0.19–1.09, *p* = 0.076)	0.81 (0.48–1.38, *p* = 0.443)	0.63 (0.36–1.12, *p* = 0.114)
Line of treatment	1	26 (33.3)	-	-	-	-
	≥3	10 (12.8)	-	-	1.39 (0.63–3.05, *p* = 0.410)	2.03 (0.89–4.64, *p* = 0.091)
	2	42 (53.8)	0.66 (0.29–1.53, *p* = 0.333)	0.58 (0.25–1.35, *p* = 0.206)	0.95 (0.45–2.01, *p* = 0.885)	0.97 (0.45–2.12, *p* = 0.945)
N. metastatic sites	≥3	42 (53.8)	-	-	-	-
	1–2	36 (46.2)	0.62 (0.26–1.48, *p* = 0.282)	0.64 (0.26–1.58, *p* = 0.334)	0.55 (0.33–0.94, *p* = 0.029)	0.55 (0.31–0.96, *p* = 0.035)
LIPI score	Low	28 (35.9)	-	-	-	-
	High-intermediate	50 (64.1)	2.37 (0.87–6.42, *p* = 0.091)	2.76 (0.98–7.82, *p* = 0.055)	2.68 (1.50–4.79, *p* = 0.001)	3.22 (1.71–6.07, *p* < 0.001)

## Data Availability

The datasets generated during and/or analyzed during the current study are available from the corresponding author on reasonable request.
